# Kamin Blocking Is Associated with Reduced Medial-Frontal Gyrus Activation: Implications for Prediction Error Abnormality in Schizophrenia

**DOI:** 10.1371/journal.pone.0043905

**Published:** 2012-08-31

**Authors:** Paula M. Moran, Jennifer L. Rouse, Benjamin Cross, Rhiannon Corcoran, Martin Schürmann

**Affiliations:** 1 School of Psychology, University of Nottingham, Nottingham, United Kingdom; 2 Division of Psychiatry, School of Community Health Sciences, University of Nottingham, Nottingham, United Kingdom; Utrecht University, The Netherlands

## Abstract

The following study used 3-T functional magnetic resonance imaging (fMRI) to investigate the neural signature of Kamin blocking. Kamin blocking is an associative learning phenomenon seen where prior association of a stimulus (A) with an outcome blocks subsequent learning to an added stimulus (B) when both stimuli are later presented together (AB) with the same outcome. While there are a number of theoretical explanations of Kamin blocking, it is widely considered to exemplify the use of prediction error in learning, where learning occurs in proportion to the difference between expectation and outcome. In Kamin blocking as stimulus A fully predicts the outcome no prediction error is generated by the addition of stimulus B to form the compound stimulus AB, hence learning about it is “blocked”. Kamin blocking is disrupted in people with schizophrenia, their relatives and healthy individuals with high psychometrically-defined schizotypy. This disruption supports suggestions that abnormal prediction error is a core deficit that can help to explain the symptoms of schizophrenia. The present study tested 9 healthy volunteers on an f-MRI adaptation of Oades' “mouse in the house task”, the only task measuring Kamin blocking that shows disruption in schizophrenia patients that has been independently replicated. Participant's Kamin blocking scores were found to inversely correlate with Kamin-blocking-related activation within the prefrontal cortex, specifically the medial frontal gyrus. The medial frontal gyrus has been associated with the psychological construct of uncertainty, which we suggest is consistent with disrupted Kamin blocking and demonstrated in people with schizophrenia. These data suggest that the medial frontal gyrus merits further investigation as a potential locus of reduced Kamin blocking and abnormal prediction error in schizophrenia.

## Introduction

One of the main ways in which environmental stimuli are selected for attention and subsequent learning is on the basis of what is already known about them. Kamin blocking is an associative learning phenomenon first shown in rats [1]. It demonstrates that stimuli can be attended to, and thus selected for learning, based on their reinforcement history. Kamin Blocking is seen where prior association of a stimulus A with an outcome blocks subsequent associative learning between the same outcome and an added stimulus B, when it is later presented in a compound AB associated with the same outcome. This effect is seen in animals and humans and can be demonstrated in a variety of learning paradigms. One explanation of the phenomenon is that prior learning about stimulus A in phase 1 ‘blocks’ learning about the second stimulus B in phase 2 because no mismatch between what is expected and the outcome (i.e. prediction error) is generated [2].

Schizophrenia patients show a reliable reduction in Kamin blocking, suggesting abnormal use of prediction error for learning [3], [4], [5], [6], [7], [8]. It has been suggested that schizophrenia symptoms such as delusions, may originate in a generalised inability to attribute salience or associability appropriately. This results in spurious prediction errors being generated which in turn leads to inappropriate associations between elements of experience in the environment and delusional thoughts are formed to respond to this [6], [9], [10], [11], [12]. There have been several independent demonstrations of abnormal Kamin blocking in people with schizophrenia, their relatives and individuals high in psychometrically-defined schizotypy [3], [8], [13], [14].

Surprisingly few studies have investigated the biological substrate of Kamin blocking despite its theoretical and clinical significance. However evidence drawn from a diverse range of experimental approaches suggests that midbrain structures may be important. The indirect dopamine agonist, amphetamine has been shown to disrupt Kamin blocking in rats [15], [16], [17]. Amphetamine infusion directly into the nucleus accumbens has also been shown to disrupt Kamin blocking suggesting that amphetamine acts through this region. Electrophysiological studies in primates have shown that the development of Kamin blocking is mirrored by reduced dopaminergic neuronal firing in the ventral tegmental area which projects to the basal ganglia, in blocking versus control groups [18], [19].

fMRI studies in healthy human volunteers indicate that performance on tasks measuring prediction error are associated with activation of the ventral striatum/nucleus accumbens. Rodriguez et al. [20] and Tobler et al., [21] using fMRI in healthy participants found that areas in the ventral putamen correlated with the prediction error signal. Tobler et al., [21] used a classic Kamin blocking design where correct responses to visual stimuli were rewarded with fruit juice.

There is also diverse evidence suggesting that frontal cortical regions may be important for Kamin blocking. Lesion of the frontal cortex in rats had been shown to abolish Kamin blocking using a conditioned avoidance paradigm [22]. Neuropsychological studies showing associations between performance on Oades' Kamin blocking task and a variety of neuropsychological tasks showed associations with tasks associated with cingulate and parietal regions [23]. Retrospective revaluation is an effect similar to Kamin blocking. It refers to the revaluation of the salience of a conditioned stimulus (CS) following newly acquired information. The perceived predictive ability of a cue can be altered after initial compound training with that cue either by training the other cue of the compound as a valid predictor of the outcome (backward blocking) or by extinguishing it (unovershadowing). These are therefore instances of retrospective revaluation. Corlett et al., [9] studied two forms of retrospective revaluation: backward blocking and unovershadowing to show event-related activation in the right prefrontal cortex which had the characteristics of a prediction error signal [24].

In our study we aimed to investigate the neural basis of Kamin blocking using a task that is replicably disrupted in schizophrenia [6] and which we have shown to correlate negatively with schizotypal personality in a non-clinical sample [5]. We investigated Kamin blocking related activation using fMRI in a tailored setup with an in-scanner Kamin blocking task, combining test session with fMRI data acquisition and learning sessions without fMRI acquisition. Analysis of fMRI data was restricted to a single large anatomically defined region of interest (ROI). On the basis of [9] and [21] this ROI comprised bilateral superior frontal gyrus (including medial, dorsolateral, orbital, and medial orbital parts), middle frontal gyrus, cingulate regions (anterior cingulate and paracingulate gyri), caudate nucleus, and putamen. The ROI also comprised supplementary motor regions (previously shown to be activated by error responses and error related feedback [25]). We identified a region in the medial prefrontal cortex portion of this ROI where - across participants - brain activation in a Kamin blocking-related contrast increased as behavioural Kamin blocking decreased.

## Materials and Methods

### Participants

18 participants, all students or staff of the University of Nottingham, participated in the experiment with full written informed consent and Nottingham University Medical School ethics committee approval. None of the participants reported neurological disease or a history of mental health history in their immediate family. Consistent with previous reports a number of participants (50%) did not demonstrate the Kamin blocking effect [3], [5], [6].This proportion was higher than in previous studies in controls which is typically 10–20%. For the present report, fMRI data analysis was restricted to participants who demonstrated Kamin blocking (see below for definition of Kamin blocking score). Nine participants (4 male, 5 female) met this criterion, mean age 22.8 years (SD 5.1, range 19–34 years). We measured psychometrically defined schizotypy in individuals prior to scanning using the Oxford –Liverpool Inventory of feelings and experience (O-LIFE) [26], as reported previously [5], comprising scales for unusual experiences (UNEX) introvertive anhedonia (INTAN), and cognitive disorganisation (COGDIS). Mean (standard deviation) O-LIFE scores for whole sample (n = 18) were as follows; UNEX: 4.17 (4.7), INTAN: 4.28(2.4), COGDIS: 7.56 (5.6). There were no significant differences in age, sex or schizoptypy scores between fMRI-evaluated and non-evaluated participants. O-LIFE scores for included participants (n = 9) were UNEX: 3.9 (4.3), INTAN: 3.8(4.3), COGDIS: 7.11 (5.5) and excluded participants (n = 9) UNEX: 4.4 (5.3), INTAN: 4.67(2.6), COGDIS: 8 (6).

### Stimuli and task

**Figure 1:**
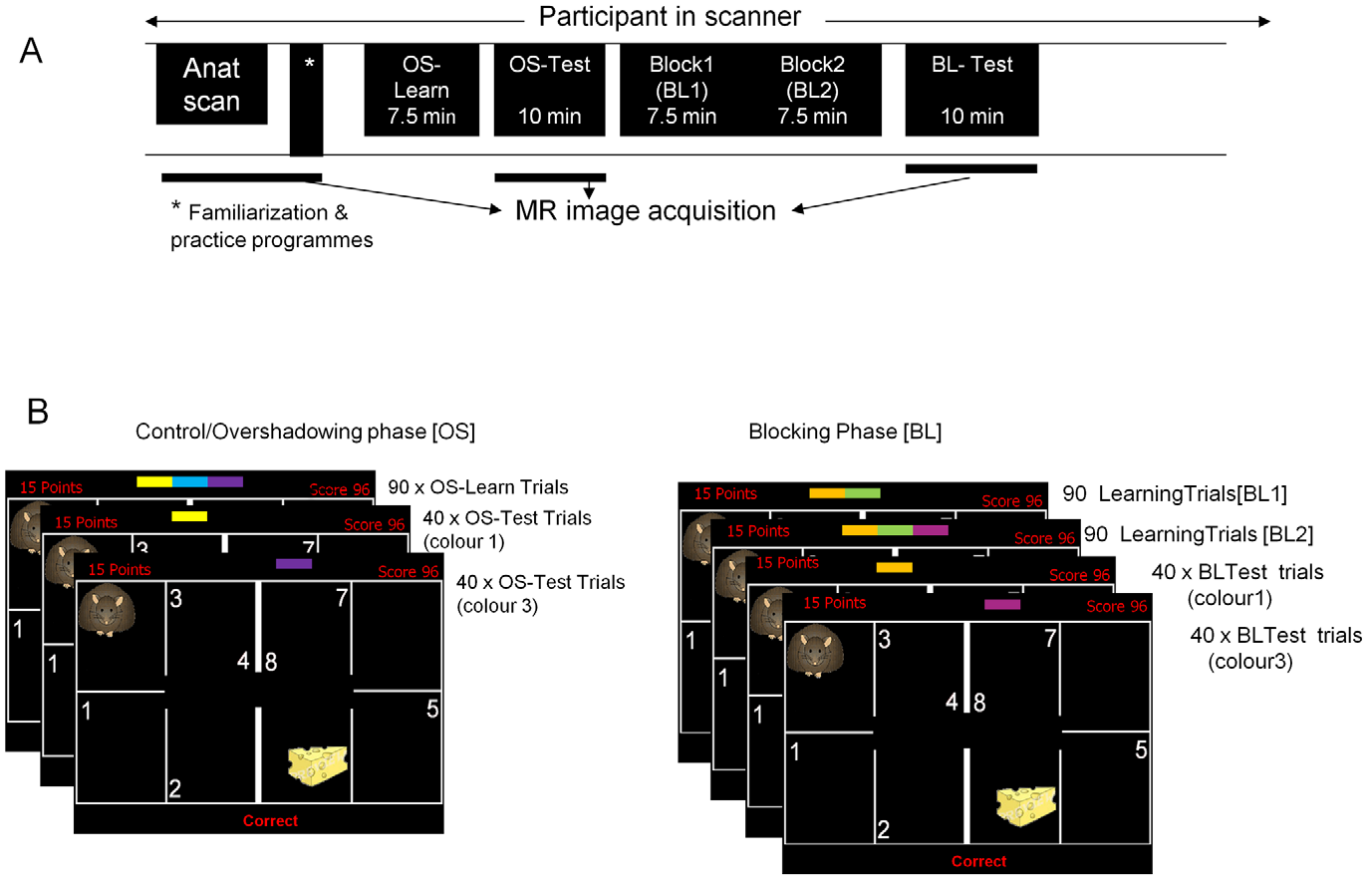
Order of conditions in the experiment (A) and outline of “mouse in the house” task (B). Anat  = anatomical scan. See methods and information S1 & S2 for more detailed explanation.

**Figure 2:**
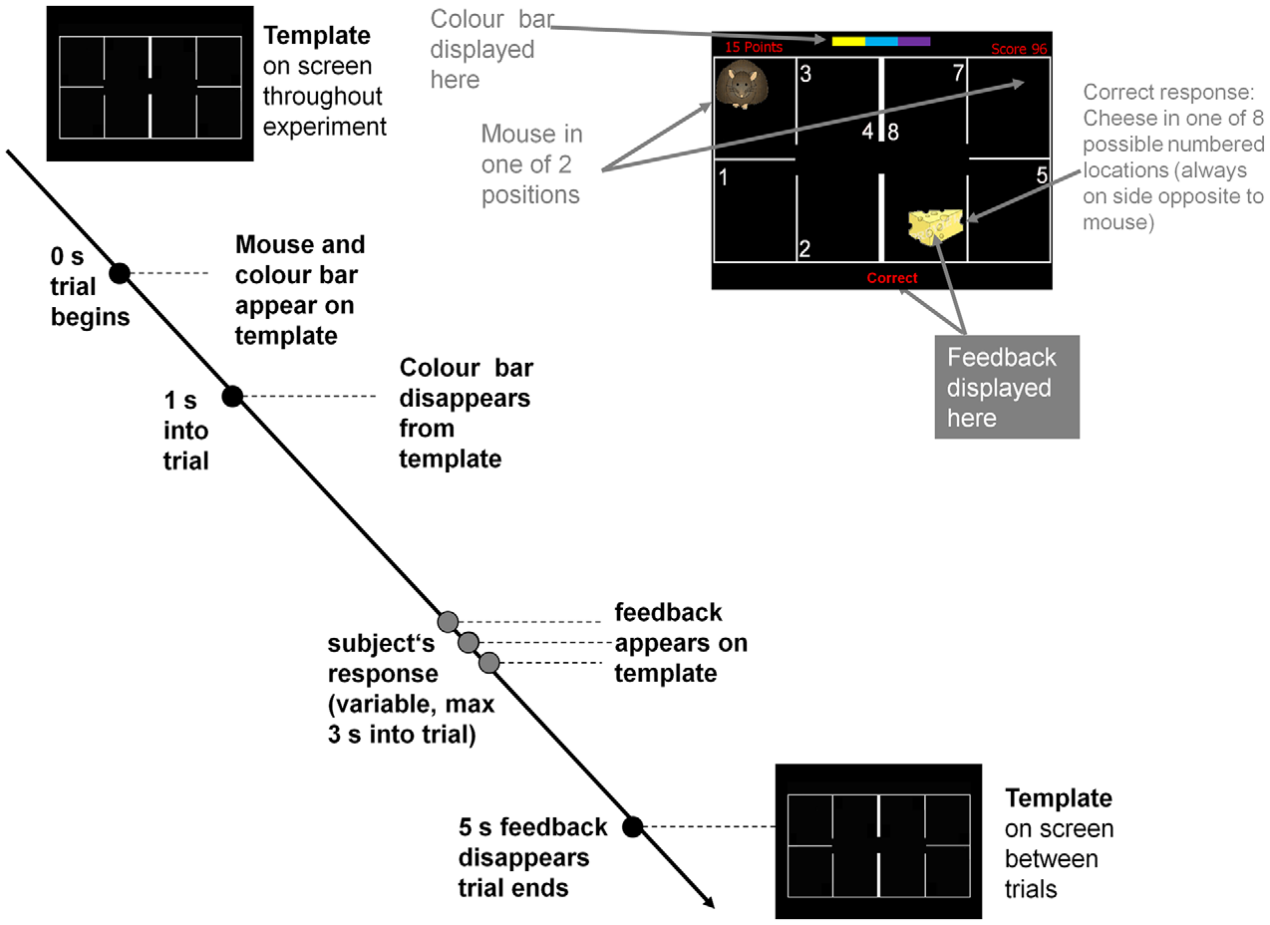
Trial structure during all sessions (OS-learn, OS-test, BL-learn, and BL-test). The “Mouse in the House” example screen (top right) refers to a dicolour-bar trail from the BL-learn condition. At the beginning of the trial, a colour bar appears near the top of the screen, and the mouse appears either left or right on the house template. After the subject's response or at 3 s into the trial (whichever is earlier), a feedback display is shown, and in case of a correct answer only, a wedge of cheese appears at any of the 8 numbered positions. See information S1 & S2 for more detailed explanation.

These were derived from Oades' behavioural experiment described in [3], [4] [5], [6] and rewritten in E-Prime V1.1 (Psychology software tools Inc.). Participants are instructed that they are a hungry mouse trying to find his cheese in a “house” (Figure 1 and 2 and Information S1 and S2 for full description of task and procedure). On a typical trial one of two sets of tri- or di- coloured rectangular bars appears on the screen for 1 second. There are two sets of colours (e.g. set 1 =  red (colour 1), green (colour 2) and blue (colour 3); or set 2 = yellow (colour 1), turquoise (colour 2) and brown (colour 3). Each set of colours corresponds to a particular location in the “house” which are numbered 1 to 8. Participants identify the location of the cheese by pressing one of two button boxes corresponding to the 8 possible locations. If the answer is correct a piece of cheese appears and “Correct” appears below the house plan, the participant earns points displayed at the top left of the screen which are added to a tally of cumulative points accrued on the upper right of the screen. If an incorrect choice is made “incorrect” is displayed on the bottom of the screen or there a failure to respond within the period then “no response detected” is displayed.

The present study used an fMRI-adapted version of Oades' task (1996) as an in-scanner Kamin blocking task (see Information S1 for full details). At the beginning of each fixed length 5-s trial, mouse and colours were superimposed on the house template (the template remained on screen throughout the fMRI experiment, except for breaks between sessions). At a time of 1 s into the trial, the colours disappeared. Subjects were instructed to press one out of eight response buttons (4 on each hand) as quickly and accurately as possible. Once a button press was detected (or at 3 s into the trial, whichever was earlier), feedback information was superimposed on the house template. At the end of the trial (5s after onset), feedback information disappeared. The next trial followed either immediately or after 1, 2, or null trials (5 – 15 s with only house template on screen). Test trials comprised 40 trials of colour 1 and 40 trials of colour 3, resulting in a total duration of 120*5 s = 10 min (inclusive of 40 null trials).

The KB task is run in two conceptual phases 1) Overshadowing (OS) with a test phase and 2) Blocking (BL) with a test phase. In OS there were 90 training trials where blocks of three colours were presented. This number of trials was determined on the basis of pilot behavioural experiments such that 100% of participants showed learning. Test trials (described above) probed how much learning had accrued to individual elements of the tri-colour blocks. In BL there were 90 training trials with a di-colour bar followed by 90 trials where an additional colour was added to form a tri-colour bar. These sessions were followed by test trials probing how much learning had accrued to individual elements of the tri-colour blocks. As training with the two colour bar fully predicts the spatial location, blocking should be demonstrated as slower or no learning about the third added colour. A Kamin blocking score was calculated from the mean difference in latency to respond to the first and third colour bars in the two conditions OS and BL: Reaction times (RT) are calculated in milliseconds (ms).
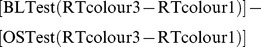



This calculation of Kamin blocking as a function of an overshadowing/basal learning condition subtracts any potential confounding influence of overshadowing, colour, laterality of stimulus presentation etc. on reaction times. This blocking score was calculated for each of the test trial pairs and the latency difference was averaged across pairs of trials. Data from the first trial pair were discarded to remove any confounds of the effect of surprise when trial format changed to individual presentation as in previous studies [3] [5], [6]. The task began once anatomical scanning was complete. Subjects remained in the scanner from the beginning of the anatomical scan to the end of BL-test, altogether approximately 65 min.

A button-box practice programme and a familiarisation programme (details in information S1) were run during the anatomical scan to familiarise participants with the set-up prior to the Kamin blocking protocol, summarised below (see Figure 1 for experiment overview):

- **1] OS- Learning** [90 trials (tri-colour bar) @5s each =  450s, 7.5 min).
- **2] OS- Test** (120 trials (single colour bar), 40 trials colour 1, 40 trials colour 3, 4 null trials @ 5s each =  600 secs, 10 min.
- **3] BL-Learning** separated into 90 trials (di-colour bar) then 90 trials (tri-colour bar comprised of di-colour bar with extra coloured rectangle added @5 secs each, 2×450s, 2×7.5 min).
- **4] BL-Test** (120 trials (single colour bar), 40 trials colour 1, 40 trials colour 3, 4 null trials @ 5s each =  600 secs, 10 min.

### Scanning

MR images were acquired on a Philips Intera 3T scanner with 8-channel SENSE coil. Anatomical scans used an SPGR sequence with 1mm×1mm×1mm, resolution. 160 slices covered the whole brain. Functional scans used echo-planar imaging with TR 2.5s, TE 40 ms, FA 85°, matrix 64×64, field of view 192×192, in-plane resolution 3mm×3mm and slice thickness 3 mm (without gaps), 40 slices. During each of the 2 fMRI phases (Figure 1 for experimental overview), 252 volumes were acquired (duration of each session 630 s, comprising 2 initial trials, a sequence of 80 test trials and 40 null trials, and 4 null trials at the end).

### fMRI data evaluation

**Figure 3:**
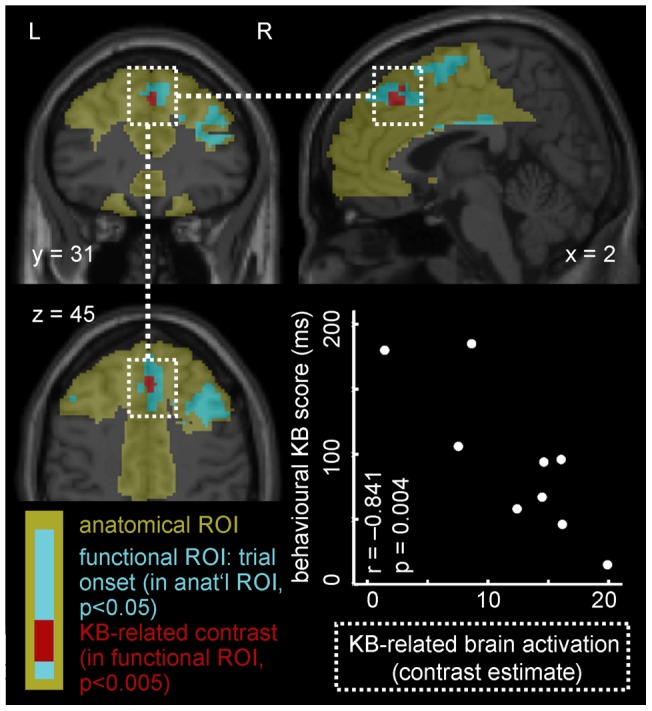
Kamin-blocking-related activation (KB, red) as identified in random effects analysis across 9 subjects (shown at p<0.005 for display purposes instead of p<0.001 as used in analysis), peak voxel at 3 30 42 in MNI coordinates, corresponding to right medial frontal gyrus in atlas brain. Anatomical ROI (yellow) comprising superior frontal gyrus (with medial, dorsolateral, orbital, and medial orbital parts), middle frontal gyrus, cingulate regions (anterior cingulate and paracingulate gyri), caudate nucleus, putamen, and supplementary motor regions, all bilateral. Functional ROI (turquoise) defined as all voxels with trial-onset-related activation (regardless of trial type, p<0.05), restricted to anatomical ROI. The functional ROI served as search volume for Kamin-blocking-related activation. Inset, lower right: Subjects' (N = 9) behavioural Kamin blocking scores plotted against Kamin-blocking-related brain activation (read as contrast estimate from peak voxel of the 5-voxel cluster with p<0.001).

Functional data underwent standard preprocessing in SPM5 (Wellcome Trust Centre for Neuroimaging, University College London): realignment for movement correction, normalization to SPM templates (in MNI space), and spatial smoothing (6 mm×6 mm×6 mm full-width-at-half-maximum, i.e., twice the original voxel size in each dimension). For subject-level statistics, pre-processed images were entered into a general linear model with regressors representing the onsets of colour bar presentation trials. Realignment parameters were included as nuisance variables. For each subject, two contrast images were computed: (1) “Trial onset” for all colour bar presentation trials, regardless of trial type, used for masking purposes only and (2) “KB effect contrast”, [Blocking phase (colour 3- colour 1)]-[Overshadowing control phase (colour 3- colour 1)], as the contrast of interest (note the difference calculation is the same as for the behavioural KB score). For each contrast, 9 subjects' contrast images underwent random-effects group analysis. A region of interest (ROI or search volume for KB effect) was defined as the overlap of the following two volumes (shown in Figure 3): (1) a single large hypothesis-derived, anatomically defined ROI comprising superior frontal gyrus (with medial, dorsolateral, orbital, and medial orbital parts), middle frontal gyrus, cingulate regions (anterior cingulate and paracingulate gyri), caudate nucleus, putamen, and supplementary motor regions, all bilateral (based on parcellation according to [27], and (2) all voxels activated in the contrast “Trial onset”, thresholded at an extremely liberal p<0.05 uncorrected for masking purposes. The resulting ROI (search volume for KB effect) comprised 1766 voxels  = 48 ml. Within this ROI, KB-effect related activation was thresholded at a standard p<0.001 uncorrected at voxel level and at p<0.05 at cluster level with small-volume correction for the 48-ml KB-effect ROI. Locations of activated clusters are reported in MNI coordinates. Corresponding brain regions were identified in a brain atlas [28] after conversion of coordinates (mni2tal procedure, Matthew Brett, http://www.mrc-cbu.cam.ac.uk/Imaging/Common/mnispace.shtml).

## Results

### Behavioural experiment

Out of 18 participants 9 did not demonstrate Kamin blocking in this sample, the scores of these participants were in fact highly negative suggesting that an alternative strategy may have been used such as augmentation. We therefore analysed FMRI data from the remaining 9 participants that showed a Kamin blocking score >0. These were significantly greater than zero, one-sample T(8) = 4.92, P<0.001 Vs zero. Schizotypy scores did not correlate significantly with KB scores following Bonferroni correction in this sample, however INTAN was negatively correlated with KB score in the full sample (n = 18), r(18)  = −45, *P*<0.05 which is consistent with previous reports [4].

### fMRI experiment

In random effects analysis across N = 9 subjects, statistically significant brain activation in the Kamin-blocking-related contrast within the frontal-cingulate-caudate-putamen ROI was observed in a single frontal midline cluster (Fig. 3). In this 5-voxel cluster, activation exceeded a voxel-level threshold of p<0.001 and reached p = 0.030 at cluster level (with small-volume correction for the ROI). The location of the peak voxel (Zmax  = 3.77, at 3 30 42 in MNI coordinates or 3 31 37 in Talairach coordinates) corresponds to the right medial frontal gyrus portion of the frontal-cingulate-caudate-putamen ROI – Brodmann area 8 in the atlas brain.

In the second analysis step, Kamin-blocking-related contrast estimates (for the frontal midline peak voxel identified in the first step) were read subject by subject. These contrast estimates were inversely correlated with individual subjects' in-scanner behavioural Kamin Blocking scores (Pearson's r = −0.841, p = 0.004, see inset in Figure 3). Note that the first analysis step tests brain activation against zero, whereas only the second step tests for correlation between brain activation and behavioural Kamin blocking score.

## Discussion

This study aimed to identify a brain signature of Kamin Blocking using a task known to be disrupted in schizophrenia patients and healthy individuals with high psychometrically-defined schizotypy. In individuals that demonstrated the Kamin blocking effect we identified robust Kamin blocking related brain activation in right medial frontal gyrus. Across participants, this activation was negatively correlated with behavioural Kamin blocking scores.

Medial frontal gyrus is one of the regions where grey matter volume has been shown to be decreased in chronic schizophrenia, which is consistent with previous reports that this Kamin blocking task is disrupted in chronic schizophrenia patients [29]. Pomarol-Clotet et al. [29] report reduced activation in two clusters in chronic schizophrenia patients compared to controls during performance of a working memory (2-back) task, the peak of one of these clusters (MNI co-ordinates 2,26,50) is notably close to the peak identified in this study (MNI co-ordinates 3,30,42).

The medial frontal gyrus is located within the prefrontal cortical region. Kamin-blocking related activity within the prefrontal cortex is consistent with a number of previous studies investigating prediction error using associative learning paradigms [24] [30]–[32]. Roiser et al. [32] found that this activation formed a continuous relationship with aberrant learning. It has been suggested [31] that many studies investigating prediction error do not separate activation concerned with incentive value and that concerned with prediction error. As Kamin blocking is the classic prediction error design, indeed the basis of the most influential theory of prediction error [33] there is minimal interference from other learning or reward processes. Further evidence for the “pure”, primary nature of prefrontal cortical prediction error signal is also seen in Turner et al [31]. Dopamine prediction error signals can also be represented by a reduction in normal firing pattern as opposed to an increase. This reversed, or negative prediction error occurs not when the US is aversive, but rather when it occurs to a lesser extent than would be expected on the basis of previous learning [2], [20]. Turner et al.'s super-learning paradigm involves both positive and negative prediction errors. In both cases, there was event-related activation in the prefrontal cortex, suggesting that perhaps this area may be important to all forms of prediction error signalling.

It is possible therefore that areas isolated in other studies may be responsible for more specific elements of prediction error signalling. The ventral putamen, which reported by [21] is a structure in the striatum. In single-cell recording studies using primates, Schultz and Dickinson [2] concluded that neurons in this region fire in relation to unpredictability without fully recording the prediction error signal, particularly as there seems to be no negative prediction error in response to US omission. However, if this were the case, it is unclear why Tobler et al., [21] identified the ventral putamen and orbitofrontal cortex as central to prediction error. One possible explanation for this discrepancy may be the use of fruit juice as the unconditioned stimulus (US) which has a naturally appetitive value, as participants were instructed not to eat or drink for several hours before testing. It may be that natural rewards such as food, drink and sex produce prediction error activity elsewhere in the brain. Tobler et al., [21] did however find activation in many other brain areas, including the prefrontal cortex suggesting that different brain areas may be responsible for different prediction error functions.

It has been suggested that the prefrontal cortex may not be involved in early prediction error coding but rather becomes involved when inferences need to be made on the basis of prediction error. Thus it may be that tasks that have greater executive demands lead to significant prefrontal activation. This may help to explain the lack of activation in the midbrain in our study that had been expected on the basis of previous experiments; this activation may be primary. Logothetis et al.,[34] investigated the physiological basis of fMRI in primates and found that the BOLD signal was more likely to reflect neuronal input as opposed to the spiking of projection neurons. Carter et al., [35] investigated CS-US contingency awareness in classical conditioning. While activity in the amygdala was found to correlate with skin conductance measures, the implicit measure of conditioning, activity in the prefrontal cortex was found to correlate with contingency awareness. Prefrontal cortical activation has been reported to be associated with attempting to find solutions to a task with no correct answers [36] and Mamelia et al., found that activity in this area was associated with lying [37]. Deception is a commonly used paradigm to assess theory of mind which has also been associated with activity in the medial frontal gyrus and which is deficient in patients with schizophrenia [38], [39], [40], [41]. We are currently investigating whether prediction error and theory of mind abnormalities are associated, on the basis of the present data we would predict they would be.

The medial frontal gyrus has previously been associated with a number of cognitive functions that have themselves been independently associated with Kamin blocking performance such as sustained attention and uncertainty. Task-related increase in cerebral blood flow in medial frontal gyrus (among other regions) has been demonstrated in a sustained attention task [42] and a go/no-go visual reaction time task [43]. Consistent with this behavioural data from our laboratory has also indicated sustained attentional disruption in patients that have Kamin blocking deficits [44].

Medial frontal gyrus activity measured using fMRI has been associated with situations of uncertainty [45], [46] which is consistent with theoretical accounts of learning. Volz et al., [47] found positive correlation between prediction uncertainty and a number of regions including a medial frontal peak at [4,30,46 Talairach co-ordinates] which is close to that we identified in the present study [3,31,37 Talairach co-ordinates]. Kamin and later learning theorists suggested that the critical determinant of conditioning (learning) is the surprise value of the reinforcer [48]. A reinforcer attracts attention and sustains new learning when there is uncertainty about its occurrence. Once it is fully predicted the organism will no longer continue to associate other stimuli with the unconditioned stimulus (US). Volz et al., [46] suggest that there is a common cerebral correlate for uncertain predictions irrespective of whether they are internally or externally generated, but different correlates for coping strategies of uncertainty. They suggest that BA8 (corresponding to the medial frontal gyrus region of activation in this study) reflects *that* we are uncertain, while other networks reflect what we do to cope with this uncertainty. Elliott and Dolan [49] suggested that mesial BA 8 activation represents adaptive stimulus-response mappings, as distinct from internally guided guessing. Volz et al., suggest further [47] that BA 6 and mesial BA 8 are both involved in the acquisition of stimulus-response associations, but that with BA 8 specifically modulates this learning process by error evaluation. Such a possibility is supported by the present findings where we show a negative correlation with Kamin blocking. Higher Kamin blocking score considered in terms of lower prediction error generation to the added stimulus, would be reflected as lower medial frontal gyrus activation.

Further experiments would be required to ascertain the specific predictive relationship and potential temporal order of these constructs to test this hypothesis.

One limitation of this study is the small number of participants. We have adapted the “mouse in the house” programme which we know to be disrupted in people with schizophrenia and to have been independently replicated by two groups, to a design for fMRI, but a higher number of participants in the study did not show Kamin blocking compared to the behavioural trials of the task, meaning only 50% of those tested could be evaluated for Kamin blocking. It is possible that participants may have used alternative strategies to perform this task in the scanner environment. However despite this we were able to detect significant correlation between activity in medial frontal gyrus and Kamin blocking. Our sample of participants was relatively homogenous in terms of age, socio-economic status and schizotypy scores, we have no evidence that those who demonstrated Kamin blocking in this task were selectively “different” from those that did not show blocking, but this remains a possibility. Of those that did not show blocking most had extremely high negative blocking scores indicating perhaps that an alternative associative learning phenomenon such as augmentation might have occurred e.g. [50]. The reason for this requires further investigation before any firm conclusion can be drawn.

In summary we have shown that performance in the “mouse in the house” Kamin blocking task is associated with reduced activity in the medial frontal gyrus. This has implications for the neural substrate of Kamin blocking and by inference of its disruption and abnormal use of prediction error in patients with schizophrenia.

## Supporting Information

Information S1 **Adaptation of original Oades' experimental design with joystick for button box response and fMRI.** Figure 1 A: Beginning of a trial with mouse on the left B: Beginning of a trial with mouse on the right C: Trial with colour set D: Feedback showing the participant they have found the cheese.
(DOCX)

Information S2 **Experimental procedure.** Figure 2. Kamin's blocking design compared to Oades' task design.
(DOCX)
