# Supplementary material for: Kamin Blocking Is Associated with Reduced Medial-Frontal Gyrus Activation: Implications for Prediction Error Abnormality in Schizophrenia
Source: PLoS One. 2012 Aug 31;7(8):e43905. doi: 10.1371/journal.pone.0043905 (PMC3432033; doi:10.1371/journal.pone.0043905)
Supplement: Information S1 — Adaptation of original Oades' experimental design with joystick for button box response and fMRI. Figure 1 A: Beginning of a trial with mouse on the left B: Beginning of a trial with mouse on the right C: Trial with colour set D: Feedback showing the participant they have found the cheese. (DOCX) [file pone.0043905.s001.docx]

**Information S1**

*Original Oades’ experimental design with joystick adaptation for button box response and fMRI.*

0-2 seconds

At the beginning of each trial (Fig S1A or Fig S1B below), the mouse appears on the screen on the template of the house for two seconds. The location of the mouse alternates between the top left and the top right hand of the house on each trial.

The cheese is always located in the opposite side of the house the mouse is placed. In Figure S1A, the cheese is hidden under 5, 6, 7 or 8 and in Figure S1B it is hidden under 1, 2, 3 or 4. Participants are given instructions about this rule.


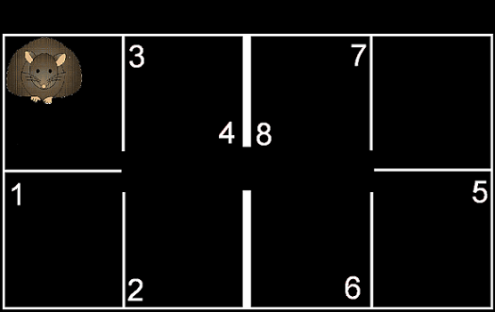


Figure S1A


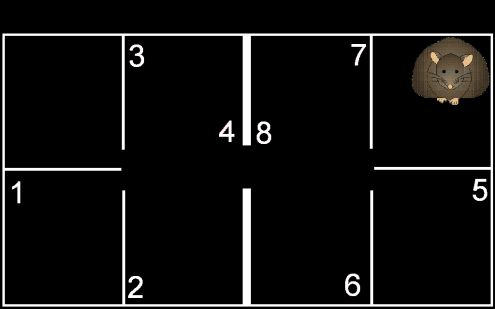


Figure S1B

2-3 seconds

Between 2 and 3 seconds, a colour set appears at the top of the screen (Figure S1C). There are two possible colour sets for the control phase of the game, and a different two possible colour sets for the blocking phase of the game. The colour sets contain either two or three colours depending on the stage of the game, as described below.

After one second, the colour disappears. Subjects are able to guess the cheese’s location from the moment the colour set first appears.


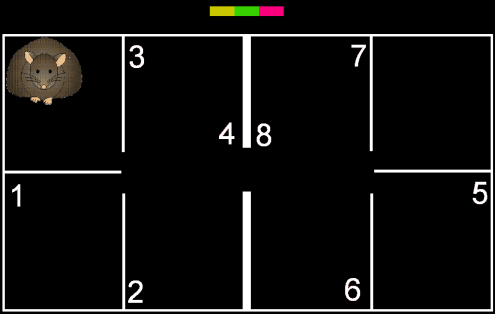


Figure S1C

2-7 seconds

The participant has a maximum of five seconds to respond. To encourage subjects to respond quickly, and to add an incentive to the task, there is a points system. If the subject takes between 0-1 seconds to respond they get 15 points, 1-2 seconds they get 12 points, 2-3 seconds 9 points, 3-4 seconds 6 points, and before the end of the 5^th^ second, 3 points. Any longer than this and the subject is rewarded 0 points and the trial ends. A counter at the top left of the screen counts down from 15 to 0 points.

As well as the top left counter, there is an incremental counter at the top right of the screen. This displays a total score, adding the number of points obtained in each trial.

Feedback Display


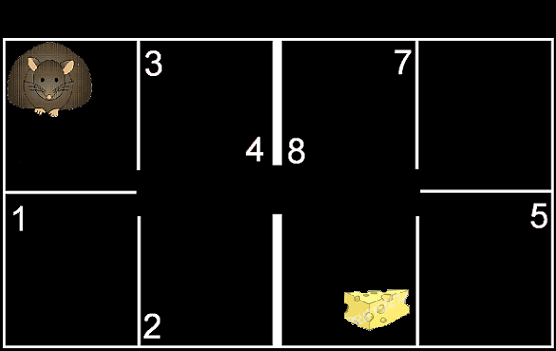


Fig S1D.

A feedback display appears immediately after response. If the response is correct a message in red text appears below the house saying “correct”, and a cheese appears in the place of the number the participant guessed (Fig S1D). If the response is incorrect, a message saying “incorrect” appears and the location of the cheese is not revealed. If the subject does not respond within the allocated period a message appears saying “no response detected” and again the correct location of the cheese is not revealed.
