# Supplementary material for: Kamin Blocking Is Associated with Reduced Medial-Frontal Gyrus Activation: Implications for Prediction Error Abnormality in Schizophrenia
Source: PLoS One. 2012 Aug 31;7(8):e43905. doi: 10.1371/journal.pone.0043905 (PMC3432033; doi:10.1371/journal.pone.0043905)
Supplement: Information S2 — Experimental procedure. Figure 2. Kamin's blocking design compared to Oades' task design. (DOCX) [file pone.0043905.s002.docx]

**Information S2**

## Experimental Procedure

The Oades task can be directly mapped onto the original blocking design by Kamin (see Fig S2 below). In the task, the coloured blocks are the conditioned stimuli and the cheese is the unconditioned stimuli. The control phase of the Oades’ task which all participants complete due to the within-subjects design, is equivalent to the control group condition of Kamin’s experiment.

Control group

Experimental group

Test

Strong Conditioned Response

**+**

🡪 SHOCK

Test

Weak Conditioned Response

**+**

🡪 SHOCK


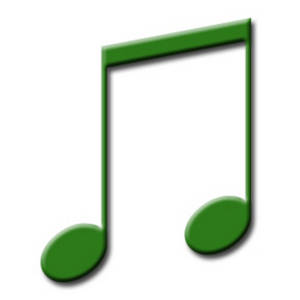

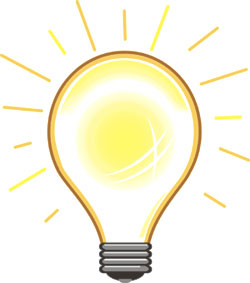

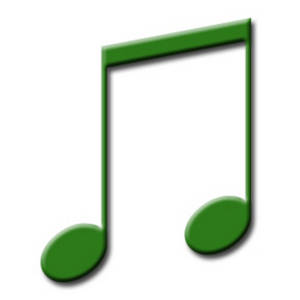

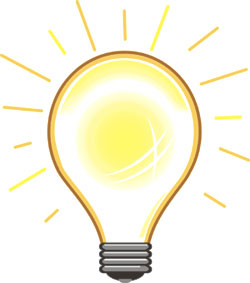

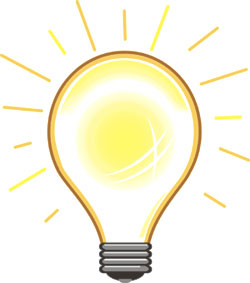

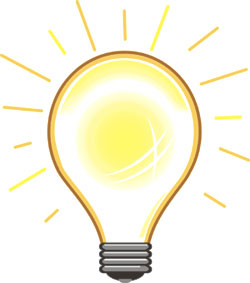


🡪 SHOCK


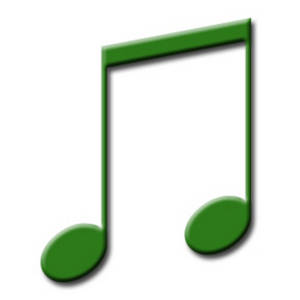


**Kamin’s Design [between subject]**

**Oades’ Task [Within Subject]**

Overshadowing control phase

Experimental phase

Test

🡪

Test

🡪

🡪


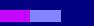

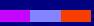

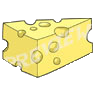

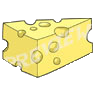

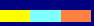

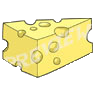

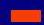

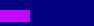

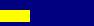

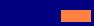


Fig. S2 Kamin’s blocking design compared to Oades’ task design

#### Overshadowing (OS) - Learning Phase

Participants were presented with three colours as in the original experiment. The middle colour is an irrelevant, or neutral stimulus. The colour on the left is conditioned stimulus 1 (CS1), equivalent to the tone in Kamin’s original experiment. The colour on the right is conditioned stimulus 2 (CS2), comparable to the light in Kamin’s experiment. Progression to the next phase depends the participant getting 11/12 trials correct.

There are two possible colour sets:

Colour set 1


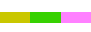


CS1 CS2

Colour set 2


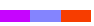


CS1 CS2

#### Overshadowing - Test Phase

In the test phase just one colour, CS1 or CS2, from either colour set was presented. Because participants had had equal exposure to CS1 and CS2, reaction times to these colours should be similar. During analysis as explained below, this data is compared to reaction time data to CS1 and CS2 in the blocking phase, where exposure to CS1 and CS2 is different.

Colour set 1


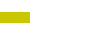


CS1


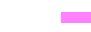


CS2

Colour set 2


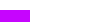


CS1


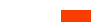


CS2

Before any presentations of CS1 or CS2, there is an individual presentation of both middle colours from each colour set. This is similar to the original Oades’ task and is intended to account for the surprise of seeing just one colour rather than the full colour set.

Stimuli are presented in a part-randomised order: CS1 and CS2 from each colour are always presented in adjacent trials, but whether CS1 or CS2 is the first trial is randomised. There are 12 presentations of CS1 and 12 presentations of CS2.

Once participants have finished the control session, a screen appears displaying their total score for the session. There is then a brief recap of instructions for participants before they proceed to the blocking session.

# 2.Blocking – learning phase

#### Learning Phase 1 (di-colour bar)

Colour set 1


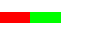


CS1

Colour set 2


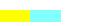


CS1

Participants are exposed to CS1 paired with the neutral colour only. Progression to next stage depends on getting at least 11/12 trials in a row correct

#### Learning Phase 2 (tri-colour bar)

The same colour sets are used as in Learning Phase 1. However, CS2 is now added. Again, progression to the test phase depends on getting at least 11/12 trials in a row correct.

Colour set 1


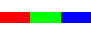


CS1 CS2

Colour set 2


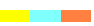


CS1 CS2

#### Test Phase

As with the Control Session Test Phase, in Blocking Session Test Phase, subjects are presented with individual colours from the colour sets and reaction times to the different colours are tested. However, as subjects are only exposed to CS2 after the association between CS1 and the cheese location is fully learnt, reaction times to CS2 should be slower if blocking has occurred.

Colour set 1


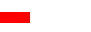


CS1


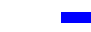


CS2

Colour set 2


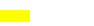


CS1


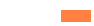


CS2

This leads to the following blocking score calculation for both colour sets:

**Experimental**

**(Reaction Time CS2 – Reaction Time CS1)**

**minus**

**(Reaction Time CS2 – Reaction Time CS1)**

**Control**


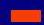

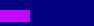

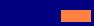

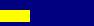


Reaction times to CS2 should be slower than reaction times to CS1 in the blocking session, if blocking has occurred. Reaction times to CS2 should be similar to reaction times to CS1 in the control session, the result of the second part of this calculation should be around zero. Thus, blocking is reflected as an overall positive score.
